# 48-Month Clinical Outcomes and Prognostic Factors in an All-Comers Population with Acute Coronary Syndrome and Chronic Coronary Syndrome Undergoing Percutaneous Coronary Intervention with a Sirolimus-Eluting Stent

**DOI:** 10.3390/jpm13111573

**Published:** 2023-11-03

**Authors:** Maciej Tyczynski, Adam Kern, Patryk Buller, Robert J. Gil, Jacek Bil

**Affiliations:** 1 Department of Invasive Cardiology, Centre of Postgraduate Medical Education, 02-507 Warsaw, Poland; maciej.tyczynski@cskmswia.gov.pl; 2 Department of Cardiology and Internal Medicine, School of Medicine, Collegium Medicum, University of Warmia and Mazury, 10-082 Olsztyn, Poland; adam.kern@uwm.edu.pl; 3 Department of Cardiology, Provincial Integrated Hospital, 09-400 Plock, Poland; p.buller@wszplock.pl; 4 Department of Cardiology, State Medical Institute of the Ministry of Interior and Administration, 02-507 Warsaw, Poland; robert.gil@cskmswia.gov.pl

**Keywords:** SES, PCI, Alex Plus, target lesion revascularization, ACS, in-stent restenosis, thin-strut stent

## Abstract

We characterized the performance as well as safety of a second-generation thin-strut sirolimus-eluting stent with a biodegradable polymer, Alex Plus (Balton, Poland), deployed in the acute coronary syndrome (ACS) setting. We enrolled patients who were subjected to percutaneous coronary intervention (PCI) between July 2015 and March 2016 and took into consideration demographics, clinical and laboratory data, and clinical outcomes. We defined the primary endpoint as the 48-month rate of major cardiovascular adverse events (MACE), including cardiac death, myocardial infarction (MI), or target lesion revascularization (TLR). The secondary endpoints were all-cause death, cardiac death, MI, and TLR rates at 12-, 24-, 36-, and 48 months. We enrolled 232 patients in whom 282 stents were implanted, including 88 ACS and 144 chronic coronary syndrome (CCS) patients. The mean age of the ACS population was 67 ± 13 years old, and 32% of it consisted of females. Patients with ACS were characterized by lower rates of arterial hypertension (85.2% vs. 95.8%, *p* = 0.004), dyslipidemia (67% vs. 81.9%, *p* = 0.01), prior MI (34.1% vs. 57.6%, *p* < 0.001), and prior PCI (35.2% vs. 68.8%, *p* < 0.001). At 48 months, among the ACS patients, the rates of MACE, death, cardiac death, MI, and TLR were 23.9%, 11.4%, 7.9%, 9.1%*,* and 10.2%, respectively. No stent thrombosis cases were reported. Multivariable Cox regression revealed that the statistically significant MACE predictors were massive calcifications in coronary arteries (HR 9.0, 95% CI 1.75–46.3, *p* = 0.009), post-dilatation (HR 3.78, 95% CI 1.28–11.2, *p* = 0.016), prior CABG (HR 6.64, 95% CI 1.62–27.1, *p* = 0.008), vitamin K antagonist use (HR 5.99, 95% CI 1.29–27.8, *p* = 0.022), and rivaroxaban use (HR 51.7, 95% CI 4.48–596, *p* = 0.002). The study findings show that Alex Plus was effective and safe in a contemporary cohort of real-world ACS patients undergoing primary PCI. The outcomes were comparable between the ACS and chronic coronary syndrome patients, with a trend of lower TLR in ACS patients at 4 years.

## 1. Introduction

The clinical spectrum of acute coronary syndromes (ACSs) comprises ST-segment elevation myocardial infarction (STEMI), non-ST-segment elevation myocardial infarction (NSTEMI), and unstable angina. These clinical scenarios are graduated, considering the disease severity and timing of their management [1]. Most ACS cases are provoked by an atherosclerotic plaque rupture accompanied by thrombus formation. Lipid-rich plaque ruptures are observed in two-thirds of ACS patients. Notably, a significant proportion of patients undergo ACS induced by plaque erosion, calcific nodules, coronary embolisms, coronary spasms, or spontaneous coronary artery dissection [2]. Many patients are high-risk patients, unstable, and often denied cardiac surgery; therefore, PCI is the only option for revascularization in this setting [3].

The incidence of STEMI has decreased in Western countries due to improved risk factor control. Nevertheless, in-hospital mortality and morbidity rates remain high [4]. Percutaneous coronary intervention (PCI) is the treatment method of choice for most ACS patients [5,6,7]. Widely used drug-eluting stents (DESs) are efficient in preventing restenosis and target lesion revascularization (TLR). However, still, there is an increased risk of neoatherosclerosis and very-late stent thrombosis, especially among ACS patients [7,8,9]. Additionally, the ruptured plaques of STEMI patients are characterized by a large necrotic core and abundant thrombi; therefore, they may be predisposed to vascular healing process impairment and an increased risk of in-stent thrombosis [10]. With advancements in DES technology made to overcome these obstacles, stents are now designed with improved alloys (cobalt–chromium or platinum–chromium), including thinner-stent struts (<80 μm), as well as enhanced polymer biocompatibility and new -limus drugs [11,12,13,14]. Stent polymers serve different aims. The polymer employed facilitates drug adhesion and release and biocompatibility and modulates thrombogenicity. Not all polymers are the same. The coating materials of stents can be divided into subgroups, such as organic or inorganic, biodegradable or durable, uniform or nonuniform drug delivery, and luminal or abluminal coating [15].

We characterized the performance and safety of PCI with a second-generation thin-strut sirolimus-eluting stent (SES) implanted in the ACS setting with a 4-year follow-up.

## 2. Materials and Methods

### 2.1. Study Design and Study Population

We collected data retrospectively, obtaining them from hospital records. We considered all consecutive patients who were subjected to PCI with sirolimus-eluting coronary stent Alex Plus (Balton, Poland) deployment between July 2015 and March 2016, as described previously [16]. We included patients with chronic coronary syndrome (CCS) as well as ACS, i.e., STEMI, NSTEMI, and unstable angina.

We took into consideration a range of baseline demographics, clinical and laboratory data, and clinical outcomes at a 48-month follow-up between CCS and ACS patients.

### 2.2. Alex plus Stent Characteristics

Alex Plus is a cobalt–chromium (L605) stent with 70 μm struts. The stent has an open-cell design with two connectors between segments. The stent releases sirolimus (1.3 μg/mm^2^) from a biodegradable polymer over the course of 8 weeks [17,18]. Alex Plus is available in the following range of parameters: diameter of 2.0–5.0 mm and length of 8.0–40.0 mm. The operator can safely overexpand the stent during post-dilatation (3.5 mm → 4.3 mm; 4.0 mm → 4.7 mm; 5.0 mm → 6.0 mm).

### 2.3. Data Collection

Hospital records allowed us to obtain data on the following comorbidities: arterial hypertension, diabetes, dyslipidemia, prior MI, prior PCI, chronic kidney disease (eGFR < 60 mL/min/1.73 m^2^), history of coronary artery bypass grafting (CABG), peripheral artery disease, prior stroke, smoking, and chronic obstructive pulmonary disease. Moreover, we analyzed procedure details, including lesion characteristics (A, B1, B2, and C according to AHA/ACC classification [19]) and periprocedural adverse events. Additionally, SYNTAX (https://syntaxscore.org accessed on 24–25 August 2023), SYNTAX II [20], and EuroScore II (https://www.euroscore.org accessed on 19–20 August 2023) parameters were calculated. We also analyzed laboratory results obtained upon admission: complete blood count with differentials (WBC—white blood cells, RBC—red blood cells, Hgb—hemoglobin, and PLT—platelets), glucose, glycated hemoglobin (HbA1c), troponin T, CK, CK-MB, lipid profiles, creatinine, and estimated glomerular filtration rate (eGFR). Finally, we provided a summary of medications prescribed upon discharge [14].

Echocardiographic data (left-ventricular ejection fraction (LVEF), left-ventricular end-diastolic diameter, posterior wall diameter, intraventricular septal diameter, tricuspid annular plane systolic excursion, and left-atrial diameter) were retrieved with a standard, commercially available diagnostic ultrasound system (iE 33, Philips Healthcare, Best, Netherlands). Measurements were obtained by experienced cardiologists and based on the European Association of Cardiovascular Imaging guidelines [21].

### 2.4. Study Endpoints

We defined the primary endpoint as the 48-month rate of major cardiovascular adverse events (MACE), including cardiac death, myocardial infarction (MI), and target lesion revascularization (TLR). The secondary endpoints were all-cause death, cardiac death, MI, and TLR rates at 12, 24, 36, and 48 months.

### 2.5. Statistical Methods

Descriptive statistics are shown as mean values with standard deviation; minimum values; median values with interquartile range; and maximum values for continuous variables, and categorical variables are presented as counts and percentages. Pearson’s Chi-squared test or Fisher’s exact test were used to compare categorical variables between 2 subgroups (CCS and ACS patients). We applied Fisher’s exact test if at least one of the subgroups had a count = 0. Wilcoxon rank sum test was performed to compare continuous variables between the 2 subgroups (CCS and ACS patients). A *p*-value < 0.05 was judged statistically significant.

Kaplan–Meier estimators with 95% confidence intervals (CI) were used to compare 48-month survival curves for various endpoints between the 2 subgroups (CCS and ACS patients). If a particular endpoint occurred for a given patient more than once in a 48-month follow-up, then survival time was treated as the time until the first occurrence of this event. Notably, when considering MACE (a composite endpoint), the survival time was defined as the period leading up to the occurrence of the first event among the following: cardiac death, MI, or TLR.

Univariable and multivariable Cox regression analyses (utilizing the Cox proportional hazards model) were conducted to assess the differences in survival rates among the groups. The multivariable Cox regression model was selected through stepwise selection, employing a backward elimination algorithm with a significance threshold set at 0.1. The outcomes, including the Hazard Ratio (HR) and corresponding 95% confidence intervals for HR, were subsequently reported.

Statistical analyses were conducted using R software version 4.2.1 (23 June 2022) (“Funny-Looking Kid”, Copyright 2022; The R Foundation for Statistical Computing Platform: x86_64-w64-mingw32/x64 (64-bit)) [14].

## 3. Results

### 3.1. Baseline Characteristics

**Figure 1:**
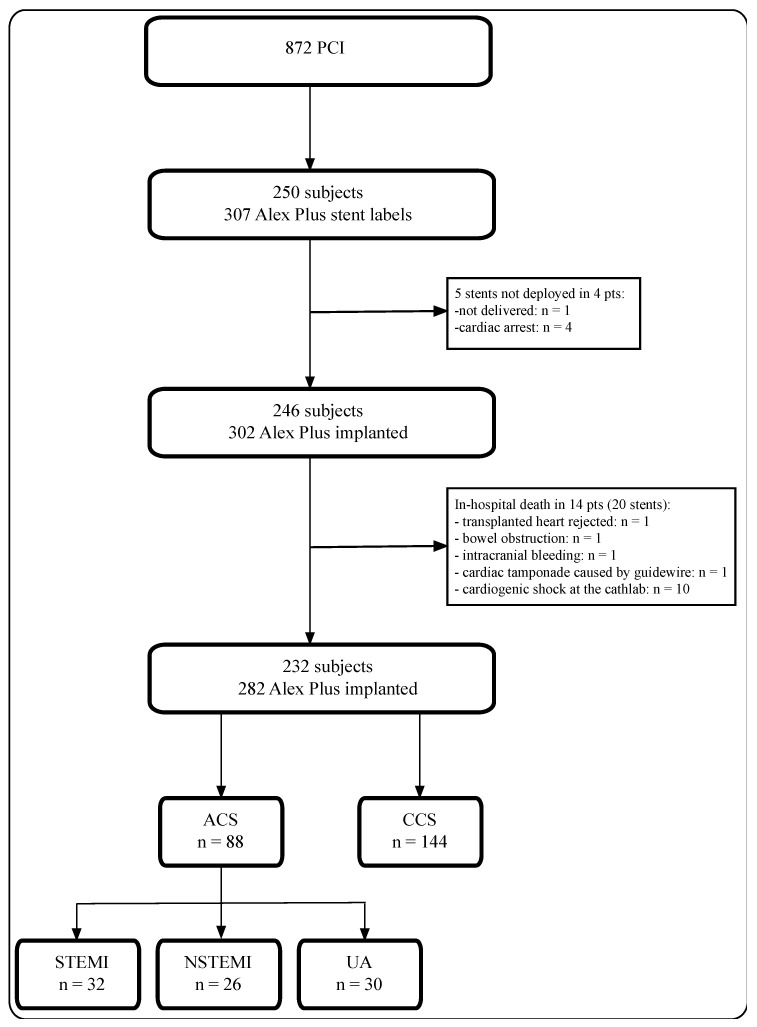
A flow chart of the study. ACS—acute coronary syndrome; CCS—chronic coronary syndrome; STEMI—ST-elevation myocardial infarction; NSTEMI—non-ST-elevation myocardial infarction; UA—unstable angina.

In the reporting time frame, we retrieved data on 872 PCI procedures. For the final analysis, we included 232 patients among whom 282 Alex Plus stents had been implanted, as described previously in detail [16]. We identified 88 patients with ACS (STEMI—32, NSTEMI—26, UA—30) and 144 patients with CCS (Figure 1).

The mean ACS population age was 67 ± 13 years old, and 32% of the subjects were females. In the ACS group, there were six cases (6.8%) of cardiogenic shock. The patients with ACS were characterized by lower rates of arterial hypertension (85.2% vs. 95.8%, *p* = 0.004), dyslipidemia (67% vs. 81.9%, *p* = 0.01), prior MI (34.1% vs. 57.6%, *p* < 0.001), and prior PCI (35.2% vs. 68.8%, *p* < 0.001) (Table 1). The patients with ACS had higher white blood cell counts and, by definition, higher levels of cardiac necrosis enzymes (Table 2).

### 3.2. Procedure Characteristics

We found no significant differences between the ACS and CCS patients, taking into consideration lesion location as well as lesion type. Mostly treated lesions were located in the right coronary artery (ACS vs. CCS: 36.4% vs. 40.3%, *p* = 0.305), followed by the left anterior descending artery (33.0% vs. 29.9%, *p* = 0.305) and left circumflex artery (26.1% vs. 26.4%, *p* = 0.305). The lesions being treated with PCI were complex. Type C lesions were treated in 37.5% of ACS cases and 40.3% of CCS cases (*p* = 0.495). Coronary bifurcations were treated in 9.1% and 10.4% of the ACS and CCS cases, respectively (*p* = 0.743). The mean SYNTAX score was higher for the ACS patients (16.0 ± 8.4 vs. 12.9 ± 8.6, *p* = 0.008) (Table 3).

In the ACS patients, transfemoral access was used more frequently (27.3% vs. 13.2%, *p* = 0.013), but there were no differences when a 6F guiding catheter was used (98.9% vs. 93.8%, *p* = 0.094). Lesions were less frequently predilated in the ACS group (53.4% vs. 66.7%, *p* = 0.043), and post-dilatations were performed at similar rates (38.6% vs. 37.5%, *p* = 0.852). The mean nominal parameters of the Alex Plus stent did not differ significantly between the groups. Device success was 100% in the ACS group and 99.3% in the CCS group (in one case, a second stent needed to be used due to heavy calcifications). Additional stents were deployed in 37.5% of the ACS cases and 39.6% of the CCS cases (*p* = 0.803). Coronary dissections were comparable between groups (5.7% vs. 7.6%, *p* = 0.568) (Table 3).

The drugs administered upon discharge are shown in Table 4. All patients received acetylsalicylic acid and P2Y12 inhibitors. In the ACS group, 88.6% of patients received clopidogrel and 11.4% received ticagrelor. ACS patients received hypoglycemic drugs less frequently (18.2% vs. 31.9%, *p* = 0.022).

### 3.3. 4-Year Outcomes

**Figure 2:**
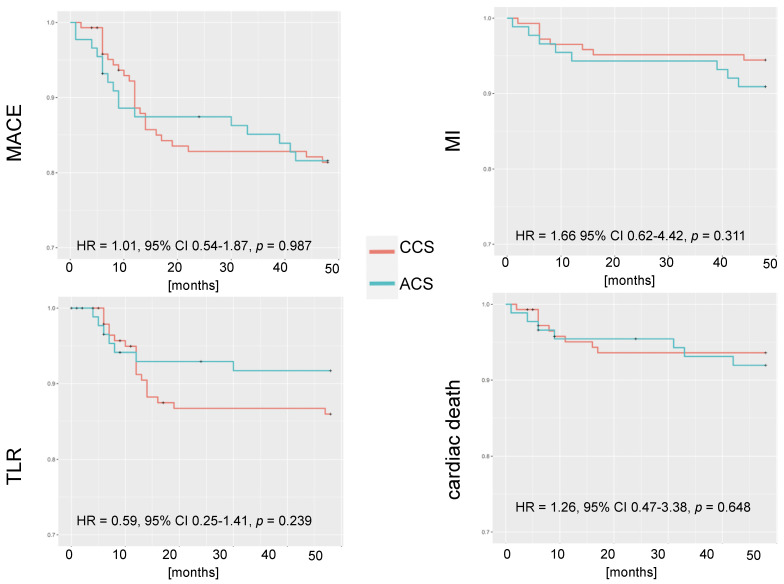
Kaplan–Meier curves regarding event-free survival in ACS and CCS subgroups. ACS—acute coronary syndrome; CCS—chronic coronary syndrome; MACE—major adverse cardiovascular events; MI—myocardial infarction; TLR—target lesion revascularization.

The incidences of MACE, death, cardiac death, MI, and TLR at 12, 24, 36, and 48 months for the whole population were published previously [16]. At 48 months, among the ACS patients, the rates of MACE, death, cardiac death, MI, and TLR were 23.9%, 11.4%, 7.9%, 9.1%, and 10.2%, respectively (Table 5). The reasons for cardiac death were heart failure deterioration (n = 5), cardiogenic shock due to MI (n = 1), and sudden cardiac death (n = 1). No stent thrombosis cases were reported. Figure 2 shows that there were no statistically significant differences between the ACS and CCS patients in terms of the analyzed endpoints at 4 years.

### 3.4. Cox Analysis

Finally, we analyzed predictive factors for MACE and TLR in the ACS subgroup at 48 months. The multivariable analyses results are depicted in Table 6 for MACE and Table 7 for TLR (univariable analyses are presented in Supplementary Tables S1 and S2).

The multivariable Cox regression revealed that the statistically significant MACE predictors were massive calcifications in the coronary arteries (HR 9.0, 95% CI 1.75–46.3, *p* = 0.009), post-dilatation (HR 3.78, 95% CI 1.28–11.2, *p* = 0.016), prior CABG (HR 6.64, 95% CI 1.62–27.1, *p* = 0.008), VKA use (HR 5.99, 95% CI 1.29–27.8, *p* = 0.022), and rivaroxaban use (HR 51.7, 95% CI 4.48–596, *p* = 0.002), whereas the TLR predictors were massive calcifications in the coronary arteries (HR 10.2, 95% CI 1.72–60.9, *p* = 0.011), second stent implantation (HR 11.8, 95% CI 1.22–114, *p* = 0.033), and a SYNTAX score of 23–32 points (HR 6.79, 95% CI 1.12–41.1, *p* = 0.037).

## 4. Discussion

This study’s findings show that Alex Plus was effective and safe in a contemporary cohort of real-world ACS patients undergoing primary PCI. PCI with Alex Plus was characterized by rare periprocedural complications and device success over 99%. The outcomes were comparable between the ACS and CCS patients, with a trend of lower TLR in the ACS patients at 4 years. This was mainly driven by the complexity of the lesions and patients. In CCS, there were more complex high-risk index procedure (CHIP) patients (higher calcification, more bifurcations, and more complex lesions).

Since the development of coronary stents in the late 1980s, constant technical and device-related improvements have been applied to diminish the rate of adverse events, especially those directly related to the stent. Contemporary DESs are designed with thinner struts (130–149 μm to 60–81 μm), and the transition in the stent platform from stainless steel to chromium alloys has decreased periprocedural and long-term complications. Thin-strut stents result in less artery injury and inflammation, thrombus formation, and neointimal proliferation than thick-strut stents [22]. Moreover, thin-strut stents are characterized by better deliverability. On the other hand, thin-strut stents might induce lower radial force and have a larger likelihood of stent deformation when advancing through tortuous anatomy. These issues highlight the significance of verifying the acquisition of results in contemporary cohorts of patients in real-world practice [23].

Some contemporary DESs release the drug from a bioresorbable polymer (BP). This offers the possibility of releasing the drug in a controlled manner and then dissolving the polymer material. This, at least theoretically, might decrease the stimulus for a persistent inflammatory state predisposing one to future ischemic events such as in-stent thrombosis [24]. In the HATTRICK-OCT trial, BP-SES enabled slightly better stent strut coverage at 3 months compared to a durable polymer (DP) zotarolimus-eluting stent (ZES) [25]. In a recent meta-analysis of patients undergoing PCI for unprotected left main coronary artery disease using ultrathin stents (with struts thinner than 81 μm), comparable outcomes in terms of MACE were observed between those treated with BP and DP stents. There were no significant variations in in-stent thrombosis between the two groups. Notably, patients with bifurcation lesions who received two BP drug-eluting stents exhibited improved results, including lower rates of MACE and target vessel revascularization (TVR). These results might suggest that minimizing persistent inflammatory stimuli is crucial in more complex and thrombogenic settings, such as those relating to ACS or bifurcation lesions [26]. The TARGET AC study revealed that patients with BP DES, after stopping dual antiplatelet therapy, exhibited a trend of lower rates of target vessel MI and ischemia-driven revascularization [27]. Nevertheless, in the most recent study, Bioflow-DAPT, no significant difference between BP and DP DES was observed [28]. Investigators assessed the effectiveness and safety of BP SES with DP ZES in high-bleeding-risk patients receiving 30-day dual antiplatelet therapy. At 12 months, the primary endpoint (cardiac death, MI, or in-stent thrombosis) was reported for 3.6% of the BP SES patients and 3.4% of the DP ZES patients (*p* < 0.0001).

Despite the all-comer nature of the study, the periprocedural complication rates in this study were low. One explanation for this phenomenon might be that transradial intervention was used in 73% of the ACS patients. Transradial access is well known to be associated with a lower risk of adverse clinical events than femoral access, particularly in MI patients [29].

At 12 months, the cardiac death, TLR, MI, and MACE rates were 4.5%, 6.8%, 4.5%, and 12.5%, respectively. The rates increased at 48 months to 7.9%, 10.2%, 9.1%, and 23.9%, respectively. These results are comparable to those reported in the literature.

Araujo et al. reported data on SESs with a strut thickness of 75 μm (Inspiron, Scitech Medical, Goiás, Brazil) vs. other contemporary DESs in STEMI patients [30]. At 17 months, the MACE rates were 14.4% for Inspiron and 16.1% for other DESs. Also, the authors reported high rates of stent thrombosis: 1.7% for Inspiron and 2.1% for other DES. Jimenez et al. compared BP SESs (Ultimaster, Terumo, Tokyo, Japan) and DP EESs (Xience DES, Abbott Vascular, Abbott Park, IL, USA) used in ACS patients from the CENTURY II study [31]. At 24 months, the cardiac death rate was 0 vs. 2.1% (*p* = 0.10), the MI rate was 2.3% vs. 4.3% (*p* = 0.38), and the TLR rate was 5.5% vs. 3.6% (*p* = 0.45). Iglesias et al. compared BP SESs (Orsiro, Biotronik AG, Baar, Switzerland) and DP EESs (Xience, Abbott Vascular, Abbott Park, IL, USA) used in ACS patients from a BIOSCIENCE study [32]. At 5 years, the all-cause death rate was 13.3% vs. 9.7% (*p* = 0.089), the cardiac death rate was 8% vs. 7% (*p* = 0.664), the MI rate was 9.9% vs. 11.5% (*p* = 0.334), the TLR rate was 9.5% vs. 9% (*p* = 0.742), and the definite stent thrombosis rate was 1.4% vs. 1% (*p* = 0.573). Tousek et al. reported data from the PRAGUE-22 study on ACS patients. In the Xience group, at 12 months, the TLR rate was 4%, and in the Magmaris group, the TLR was 12%, with a stent thrombosis rate of 4% [33].

In contrast, ACS patients from BIOFLOW-V (BP SES vs. DP EES) were characterized by very low all-cause and cardiac death (1.2–1.4% and 0–1%), TLR (3.5% vs. 3.9%, *p* = 0.823), target vessel MI (3.5% vs. 9.7%, *p* = 0.003), and MACE (7% vs. 11.9%, *p* = 0.050) rates [34]. Also, an interesting analysis was performed by Hemetsberger et al., who presnted pooled PCI results from the BIOFLOW II, IV, and V studies considering complex vs. non-complex PCI. At 3 years, target lesion failure was 14.6% for complex PCI and 8.1% for non-complex PCI (*p* < 0.001), and target vessel MI was 10.2% vs. 4.4% (*p* < 0.001), respectively [35].

Finally, we identified predicting factors of MACE and TLR. They are well known, like calcifications or higher SYNTAX scores. Nevertheless, anticoagulant use (VKA and rivaroxaban) had a strikingly high impact. This result might also be associated with the fact that 1/3 of our population consisted of high-bleeding-risk patients.

### Study Limitations

This study has some inherent limitations common to observational studies, where the treatment choice is based on the operator’s preference. The absence of randomization could potentially introduce selection bias, although this was somewhat alleviated by enrolling patients consecutively. Furthermore, the relatively small size of the study cohort and limitations in collecting follow-up data might have had an impact on the findings. Additionally, the absence of a formal calculation for the sample size could be a factor that influenced the results.

## 5. Conclusions

The study findings show that Alex Plus was effective and safe in a contemporary cohort of real-world ACS patients undergoing primary PCI. The outcomes were comparable between the ACS and CCS patients, with a trend of lower TLR in the ACS patients at 4 years.

## Figures and Tables

**Table 1:** Baseline characteristics.

Variable	Total Population N = 232 (%)	ACS N = 88 (%)	CCS N = 144 (%)	*p*
Sex: female	64 (27.6)	28 (32)	36 (25)	0.260
Age (years)	68 ± 11	67 ± 13	68 ± 9	0.788
Acute coronary syndrome type upon presentation				
Unstable angina	30 (12.9)	30 (34.2)	0	<0.001
Non-ST-elevation MI	26 (11.2)	26 (29.5)	0	
ST-elevation MI	32 (13.8)	32 (36.3)	0	
Cardiogenic shock	6 (2.6)	6 (6.8)	0	0.003
Arterial hypertension	213 (91.8)	75 (85.2)	138 (95.8)	0.004
Type 2 diabetes	97 (41.8)	32 (36.4)	65 (45.1)	0.189
Dyslipidemia	177 (76.3)	59 (67.0)	118 (81.9)	0.010
Prior myocardial infarction	113 (48.7)	30 (34.1)	83 (57.6)	<0.001
Prior PCI	130 (56.0)	31 (35.2)	99 (68.8)	<0.001
Prior CABG	22 (9.5)	7 (8.0)	15 (10.4)	0.535
Chronic kidney disease	42 (18.1)	20 (22.7)	22 (15.3)	0.153
Prior stroke	17 (7.3)	8 (9.1)	9 (6.2)	0.420
Peripheral artery disease	25 (10.8)	8 (9.1)	17 (11.8)	0.518
Chronic obstructive pulmonary disease	13 (5.6)	6 (6.8)	7 (4.9)	0.565
Echocardiographic parameters				
Left-ventricular end-diastolic diameter (mm)	50.4 ± 9.0	50.6 ± 7.3	50.2 ± 10.2	0.665
Intraventricular septal diameter (mm)	11.4 ± 2.1	11.2 ± 2.5	11.6 ± 1.8	0.197
Posterior wall diastolic diameter (mm)	10.5 ± 1.6	10.2 ± 1.8	10.7 ± 1.4	0.124
left atrium (mm)	40.4 ± 5.9	39.4 ± 5.9	41.3 ± 5.8	0.033
TAPSE (mm)	22.0 ± 4.3	21.0 ± 3.2	22.0 ± 3.8	0.609
LVEF [%]	49.5 ± 10.5	48.0 ± 11.1	50.6 ± 9.9	0.075
Severe mitral insufficiency	6 (3.1)	3 (3.5)	3 (2.8)	0.999
Severe aortic insufficiency	1 (0.5)	1 (1.2)	0 (0.0)	0.438
Severe aortic stenosis	4 (2.1)	1 (1.2)	3 (2.8)	0.633

ACS—acute coronary syndrome; CCS—chronic coronary syndrome; MI—myocardial infarction; CABG—coronary artery bypass grafting; LVEF—left-ventricular ejection fraction; PCI—percutaneous coronary intervention; TAPSE—tricuspid annular plane systolic excursion.

**Table 2:** Laboratory results.

Variable	Total Population N = 232	ACS N = 88 (%)	CCS N = 144 (%)	*p*
White blood cells (10^9^/L)	8.5 ± 2.7	9.7 ± 3.0	7.8 ± 2.1	<0.002
Hemoglobin (g/dL)	13.4 ± 1.7	13.4 ± 1.7	13.3 ± 1.6	0.861
Red blood cells (10^12^/L)	4.4 ± 0.5	4.5 ± 0.5	4.4 ± 0.5	0.477
Platelets (10^9^/L)	222.9 ± 65	226.7 ± 67.2	220.3 ± 63.5	0.580
Glucose (mg/dL)	136.4 ± 64.9	147.6 ± 72.1	125.4 ± 55.1	0.005
HbA1c (%)	6.3 (6.0–7.3)	6.3 (5.8–7.3)	6.3 (6.1–7.3)	0.592
Total cholesterol (mg/dL)	163.9 ± 50.9	170.5 ± 52.2	157.5 ± 49.0	0.067
HDL (mg/dL)	45.7 ± 14.6	46.2 ± 16.7	45.2 ± 12.3	0.764
LDL (mg/dL)	89.8 ± 40.5	94.7 ± 43.0	85.0 ± 37.5	0.135
Triglycerides (mg/dL)	142 ± 33.9	147.2 ± 126.3	137.0 ± 141.4	0.564
Creatine (mg/dL)	1.1 ± 0.7	1.2 ± 1.0	1.1 ± 0.4	0.244
eGFR (mL/min/1.73 m^2^)	70.5 ± 23.2	68.5 ± 25.9	71.9 ± 21.1	0.269
Troponin I upon admission (ng/mL)	108 (15.8–235)	281.4 (50.5–213)	44 (9.5–119.5)	<0.001
Troponin I max (ng/mL)	1110 (49.8–11,573)	5294 (688–32,561)	53.8 (14–538)	<0.001
CK at admission (IU/L)	134.5 (84–326)	232 (124–626)	85 (67–121)	<0.001
CK max (IU/L)	173 (90–473)	295 (164–963)	89 (67–152)	<0.001
CK-MB at admission (IU/L)	18 (13.5–30)	23 (17–47)	15 (12–18.2)	<0001
CK-MB max (U/L)	22.5 (15–48.5)	41 (22–116.5)	16 (13–22.8)	<0.001

Results are presented as mean ± standard deviation or median with interquartile range; ACS—acute coronary syndrome; CCS—chronic coronary syndrome; CK—creatine kinase; HDL—high-density lipoprotein; LDL—low-density lipoprotein.

**Table 3:** Periprocedural characteristics.

Variable	Total Population N = 232 (%)	ACS N = 88 (%)	CCS N = 144 (%)	*p*
Coronary artery with the target lesion				
LM	9 (3.9)	5 (5.7)	4 (2.8)	0.305
LAD	72 (31)	29 (33.0)	43 (29.9)	
LCx	61 (26.3)	23 (26.1)	38 (26.4)	
RCA	90 (38.8)	32 (36.4)	58 (40.3)	
VG	6 (2.6)	5 (5.7)	1 (0.7)	
Type of the target lesion				
A	38 (16.4)	13 (14.8)	25 (17.4)	0.495
B1	66 (28.4)	30 (34.1)	36 (25.0)	
B2	37 (15.9)	12 (13.6)	25 (17.4)	
C	91 (39.2)	33 (37.5)	58 (40.3)	
Heavy calcification	18 (7.8)	4 (4.5)	14 (9.7)	0.153
Coronary bifurcation	23 (9.9)	8 (9.1)	15 (10.4)	0.743
SYNTAX	13.9 ± 8.7	16.0 ± 8.4	12.9 ± 8.6	0.008
SYNTAX II PCI	32.9 ± 11.0	35.6 ± 10.1	31.6 ± 11.2	0.003
SYNTAX II CABG	29.1 ± 10.8	29.9 ± 10.5	28.6 ± 11.1	0.491
EuroScore II	1.6 (0.9–3.3)	2.5 (1.3–4.3)	1.3 (0.8–2.5)	<0.001
Lesion pre-dilatation	143 (61.6)	47 (53.4)	96 (66.7)	0.043
Stent diameter (mm)	3.2 ± 0.5	3.2 ± 0.5	3.1 ± 0.5	0.069
Stent length (mm)	21.2 ± 10.9	21.9 ± 12	20.8 ± 10.2	0.821
Stent pressure (atm)	15.3 ± 2.7	15.5 (2.6)	15.2 (2.7)	0.578
2nd stent implantation	90 (39)	33 (37.5)	57 (39.6)	0.803
Stent post-dilatation	88 (37.9)	34 (38.6)	54 (37.5)	0.852
Access site *				
Transradial	193 (83.2)	64 (72.7)	129 (89.3)	0.013
Transfemoral	43 (18.5)	24 (27.3)	19 (13.2)	
Guiding catheter *				
6F	222 (95.7)	87 (98.9)	135 (93.8)	0.094
7F	11 (4.7)	1 (1.1)	10 (6.9)	
Coronary dissection	16 (6.9)	5 (5.7)	11 (7.6)	0.568
MI type 4a	5 (2.2)	0	5 (3.5)	0.159

* more than one access or catheter were used during the procedure; ACS—acute coronary syndrome; CCS—chronic coronary syndrome; LM—left main; LAD—left anterior descending artery; LCx—left circumflex artery; MI—myocardial infarction; RCA—right coronary artery; VG—vein graft.

**Table 4:** Drugs administered upon discharge.

Variable	Total Population N = 232 (%)	ACS N = 88 (%)	CCS N = 144 (%)	*p*
Acetylsalicylic acid	232 (100)	88 (100.0)	144 (100.0)	-
P2Y12				
Clopidogrel	214 (92.2)	78 (88.6)	136 (94.4)	0.065
Prasugrel	1 (0.4)	0	1 (0.7)	
Ticagrelor	17 (7.3)	10 (11.4)	7 (4.9)	
Beta-blocker	223 (96.1)	84 (95.5)	139 (96.5)	0.733
Ca-blocker	53 (22.8)	15 (17.0)	38 (26.4)	0.100
Angiotensin-converting enzyme inhibitor	190 (81.9)	74 (84.1)	116 (80.6)	0.497
Angiotensin receptor blocker	36 (15.5)	10 (11.4)	26 (18.1)	0.172
Diuretic	125 (53.9)	48 (54.5)	77 (53.5)	0.874
Mineralocorticoid receptor antagonist	48 (20.7)	23 (26.1)	25 (17.4)	0.109
Nitrates	13 (5.6)	3 (3.4)	10 (6.9)	0.380
Vitamin K antagonist	17 (7.3)	8 (9.1)	9 (6.2)	0.420
Non-vitamin K oral anticoagulant	11 (4.7)	1 (1.1)	10 (7.0)	0.265
Statin	230 (99.1)	88 (100.0)	142 (98.6)	0.527
Hypoglycemic medications	62 (26.7)	16 (18.2)	46 (31.9)	0.022
Insulin	33 (14.2)	16 (18.2)	17 (11.8)	0.177

ACS—acute coronary syndrome; CCS—chronic coronary syndrome.

**Table 5:** Study endpoints arranged by year for ACS patients.

Year	Death	Cardiac Death	TLR	MI	MACE
1	5 (5.7)	4 (4.5)	6 (6.8)	4 (4.5)	11 (12.5)
2	6 (6.8)	4 (4.5)	7 (7.9)	4 (4.5)	12 (13.6)
3	8 (9.1)	6 (6.8)	8 (9.1)	4 (4.5)	15 (17.1)
4	10 (11.4)	7 (7.9)	9 (10.2)	8 (9.1)	21 (23.9)

n (%). MACE—major adverse cardiovascular events; MI—myocardial infarction; TLR—target lesion revascularization.

**Table 6:** Multivariable Cox analysis: major adverse cardiovascular events.

Variable	Multivariable Analysis for MACE		
	HR	95% CI	*p*
High bleeding risk	1.63	0.44–6.02	0.461
Calcification	9.00	1.75–46.3	0.009
Post-dilatation	3.78	1.28–11.2	0.016
Prior coronary artery bypass grafting	6.64	1.62–27.1	0.008
VKA—vitamin K antagonist use	5.99	1.29–27.8	0.022
Rivaroxaban use	51.7	4.48–596	0.002

**Table 7:** Multivariable Cox analysis: target lesion revascularization.

Variable	Multivariable Analysis for TLR		
	HR	95% CI	*p*
Calcifications	10.2	1.72–60.9	0.011
2nd stent	11.8	1.22–114	0.033
SYNTAX 23–32	6.79	1.12–41.1	0.037

## Data Availability

Data are available from the corresponding author on request.

## Supplementary Materials

The following supporting information can be downloaded at: https://www.mdpi.com/article/10.3390/jpm13111573/s1. Table S1: Univariable Cox regression for MACE; Table S2: Univariable Cox regression for TLR.
