# Supplementary material for: 48-Month Clinical Outcomes and Prognostic Factors in an All-Comers Population with Acute Coronary Syndrome and Chronic Coronary Syndrome Undergoing Percutaneous Coronary Intervention with a Sirolimus-Eluting Stent
Source: J Pers Med. 2023 Nov 3;13(11):1573. doi: 10.3390/jpm13111573 (PMC10672598; doi:10.3390/jpm13111573)
Supplement: Supplementary file 1 [file jpm-13-01573-s001.zip › jpm-2648733-supplementary.pdf]

Supplementary Tables

Supplementary Table S1. Univariable Cox regression for MACE

Characteristics	N	HR[1]	95% CI[1]	p-value
Sex	88			
Male		—	—	
Female		0.70	0.23, 2.17	0.538
Age	88	1.01	0.97, 1.05	0.726
CHIP	88	1.56	0.58, 4.20	0.374
HBR	88	2.67	0.99, 7.16	0.052
pien	88	5.30	1.51, 18.6	0.009
stent_length	88	1.02	0.98, 1.05	0.403
stent_diameter	88	1.52	0.58, 3.98	0.398
Lesion type	88			
A/B1		—	—	
B2/C		1.65	0.60, 4.54	0.332
Calcification	88	3.49	0.79, 15.4	0.100
second_stent	87	1.36	0.51, 3.66	0.539
MV_predilat	88	1.32	0.48, 3.64	0.589
MV_postdilat	88	2.29	0.85, 6.15	0.100
SYNTAX	81			
< 23		—	—	
23-33		2.09	0.46, 9.43	0.338
>= 33		0.00	0.00, Inf	0.999
SYNTAX_II_PCI	81			
<= 21.5		—	—	
21.5-30.6		3.26	0.36, 29.2	0.290
>= 30.6		2.02	0.25, 16.2	0.507
SYNTAX_II_CABG	68			
<= 21.5		—	—	
21.5-30.6		0.93	0.17, 5.09	0.935
>= 30.6		1.68	0.36, 7.92	0.510
Euroscore_II	88			
< 3		—	—	
3-5		0.55	0.12, 2.55	0.444
>= 5		1.45	0.48, 4.31	0.509
cardiogenic_shock	88	2.25	0.51, 9.90	0.284
HT	88	2.83	0.37, 21.4	0.314
DM	88	2.56	0.95, 6.89	0.062
dyslipidemia	88	8.48	1.12, 64.2	0.039
MI	88	2.17	0.81, 5.78	0.122
PCI	88	3.57	1.29, 9.83	0.014
CABG	88	3.29	0.94, 11.6	0.063
AO	88	0.72	0.09, 5.42	0.746
Stroke/TIA	88	1.53	0.35, 6.73	0.575
smoke	88	1.17	0.43, 3.23	0.755
CKD	88	1.70	0.59, 4.88	0.327

Characteristics	N	HR[1]	95% CI[1]	p-value
COPD	88	2.02	0.46, 8.92	0.351
medicine_clopidogrel	88	0.52	0.15, 1.82	0.307
medicine_ticagrelor	88	1.92	0.55, 6.75	0.307
medicine_ACEI	88	0.83	0.24, 2.92	0.776
medicine_ARB	88	1.07	0.24, 4.71	0.927
medicine_betabloker	88	0.63	0.08, 4.74	0.650
medicine_digoxin	88	9.65	1.22, 76.3	0.032
medicine_Ca_bloker	88	1.14	0.33, 4.01	0.835
medicine_diuretics	88	1.90	0.66, 5.46	0.235
medicine_MRA	88	1.26	0.44, 3.62	0.670
medicine_NTG	88	1.74	0.23, 13.1	0.594
medicine_alfa_adrenolitic	88	3.08	0.69, 13.7	0.140
medicine_VKA	88	3.97	1.27, 12.4	0.018
medicine_rivaroxaban	88	18.6	2.16, 160	0.008
medicine_LMWH	88	1.82	0.24, 13.8	0.561
medicine_IPP	88	0.76	0.17, 3.33	0.714
medicine_hypoglycaemic drug	88	2.21	0.77, 6.37	0.141
medicine_insulina	88	0.69	0.16, 3.03	0.622
echo_EF	86	0.98	0.94, 1.02	0.260

[1]HR = Hazard Ratio; CI = Confidence Interval.

Supplementary Table S2. Univariable Cox regression for TLR

Characteristics	N	HR[1]	95% CI[1]	p-value
Sex	88			
Male		—	—	
Female		1.55	0.35, 6.91	0.568
Age	88	1.00	0.94, 1.05	0.892
CHIP	88	7.37	0.89, 61.3	0.064
HBR	88	1.60	0.36, 7.16	0.537
pien	88	3.98	0.48, 33.1	0.201
stent_length	88	1.05	1.01, 1.10	0.008
stent_diameter	88	0.86	0.18, 4.01	0.846
Lesion type	88			
A/B1		—	—	
B2/C		5.68	0.68, 47.1	0.108
Calcification	88	8.85	1.71, 45.9	0.009
second_stent	87	4.41	0.85, 22.7	0.076
MV_predilat	88	4.86	0.58, 40.4	0.143
MV_postdilat	88	2.08	0.47, 9.31	0.336
SYNTAX	81			
< 23		—	—	
23-33		5.72	1.05, 31.3	0.044
>= 33		0.00	0.00, Inf	0.999
SYNTAX_II_CABG	68			

Characteristics	N	HR[1]	95% CI[1]	p-value
<= 21.5		—	—	
21.5-30.6		0.46	0.03, 7.33	0.581
>= 30.6		1.62	0.18, 14.5	0.668
Euroscore_II	88			
< 3		—	—	
3-5		0.65	0.07, 5.77	0.695
>= 5		1.43	0.26, 7.79	0.681
cardiogenic_shock	88	2.64	0.32, 21.9	0.369
DM	88	1.54	0.34, 6.89	0.572
MI	88	5.31	1.03, 27.4	0.046
PCI	88	5.19	1.01, 26.8	0.049
CABG	88	2.91	0.35, 24.2	0.323
stroke	88	2.00	0.24, 16.6	0.521
smoker	88	0.94	0.21, 4.20	0.936
CKD	88	1.59	0.31, 8.18	0.581
COPD	88	2.15	0.26, 17.9	0.478
medicine_clopidogrel	88	0.81	0.10, 6.75	0.848
medicine_ticagrelor	88	1.23	0.15, 10.2	0.848
medicine_ACEI	88	0.24	0.03, 2.00	0.187
medicine_ARB	88	0.80	0.10, 6.68	0.840
medicine_betabloker	88	2.24	0.43, 11.5	0.336
medicine_digoxin	88	1.09	0.21, 5.62	0.918
medicine_Ca_bloker	88	3.31	0.39, 28.0	0.271
medicine_diuretics	88	1.74	0.21, 14.5	0.607
medicine_MRA	88	0.67	0.08, 5.54	0.708
medicine_NTG	88	1.86	0.36, 9.60	0.458
medicine_alfa_adrenolitic	88	0.83	0.10, 6.92	0.866
echo_EF	86	1.01	0.93, 1.09	0.831

[1]HR = Hazard Ratio, CI = Confidence Interval
